# The impact of digital economy on green total factor productivity considering the labor-technology-pollution factors

**DOI:** 10.1038/s41598-023-50400-0

**Published:** 2023-12-21

**Authors:** Yipeng Huang, Zhiguo Chen, Huiru Li, Shi Yin

**Affiliations:** 1 School of Economics, Hebei University, Baoding, 071000 China; 2 Research Center for Resource Utilization and Environmental Protection, Hebei University, Baoding, 071000 China; 3 College of Economics and Management, Hebei Agricultural University, Baoding, 071000 China

**Keywords:** Environmental sciences, Environmental social sciences

## Abstract

The digital economy provides new impetus for the high-quality development of manufacturing industry. Through the DEA-Malmquist model and panel regression model, this paper confirmed that there is a positive and significant relationship between the development of digital economy and the green total factor productivity (GTFP) of manufacturing industry. The research result is as follows: (1) the development of digital economy can enhance the overall GTFP of manufacturing industry. (2) The green technology progress brought by the development of digital economy is the main path to promote the GTFP of manufacturing industry. (3) The heterogeneity analysis shows that the impact of digital economy on GTFP of high pollution manufacturing industry is significantly positive, the impact of labor-intensive manufacturing industry is significantly negative, and the impact of technology intensive manufacturing industry is not obvious. The contributions of this study are as follow. In terms of theory, this study theoretically continues Solow’s classical theory, demonstrating the scientific nature of digital technology progress in promoting GTFP growth. In empirical analysis, this study build a new digital economy development level evaluation index system based on the perspective of manufacturing industry. In addition, this study also add a labor-technology-pollution perspective for the development of relevant policies.

## Introduction

At present, the fourth industrial revolution is globally emerging, and its signature product is the digital economy, which is based on digital production processes, robotic equipment, digital services, and connection of the virtual digital world with the real world, which is typically manifested by the deep integration of data elements, cyber-physical production systems, and the Internet of Things (IoT)^1,2^. At a time when China's economy is turning toward an important historical period of high-quality development, the digital economy, a brand-new economic form, is increasingly becoming an important force for promoting economic transformation and realizing green production^3,4^. The development of China's digital economy has already surpassed that of Europe, and its potential for future development is even greater^5^. Along with the development of the digital economy, the manufacturing industry, which is an important part of China's national economy, has also embraced new opportunities for green development^6^.

Existing studies have found that the digital economy has a positive impact on total factor productivity^7^. Yang and Zeng^8^ analyzed the impact and mechanism of the digital economy on regional total factor productivity in China; they found that the digital economy promotes the development of regional total factor productivity on the whole, and technologically developed regions are more able to take advantage of the digital economy. Hu et al.^9^ explored the spatial correlation between the digital economy and regional total factor productivity by utilizing 10-year data from 30 provinces in China, and their conclusions were consistent with those of Yang and Zeng^8^, in addition, they found that the digital economy has obvious spatial spillover effects^9^. Guo et al.^10^ explored the impact and mechanism of the digital economy on the total factor productivity of enterprises based on data on cities and some listed companies in China, and they found that state-owned enterprises and large-scale enterprises responded more positively to the digital economy. Yu et al.^7^ found that the total factor productivity of manufacturing enterprises significantly increased after e-commerce interventions. Wang et al.^11^ found that the digital economy has a nonlinear inverted-U-shaped relationship with total factor productivity in the manufacturing industry by using data from Chinese A-share companies. On the other hand, Dong et al.^12^ constructed a set of mathematical and theoretical models to analyze the impact of the digital economy on the growth of FDI. Based on the perspective of blockchain technology, Xu et al.^13^ found that it also had a significant and positive impact on the total factor productivity of enterprises.

As an important indicator for measuring high-quality economic development, green total factor productivity not only considers inputs and outputs in the traditional sense, but also takes the impacts of resource consumption and pollution emissions into account; thus, it can comprehensively reflect the dual effects of production efficiency and environmental protection^14–16^. This kind of index allows individuals from all walks of life to more comprehensively and objectively assess the quality and efficiency of economic activities, and it can help the state and industrial managers to formulate economic policies and industrial management strategies more scientifically so as to promote the sustainable development of China's economy. Therefore, an in-depth investigation of the impact of the digital economy on green total factor productivity in the manufacturing industry is of great practical significance for promoting the synergistic development of the digital economy and ecological economy. Zhu et al.^17^ argued that the digital economy has a greater effect on the enhancement of green total factor productivity in the central and western regions, and it has a spatial spillover effect. Cheng et al.^18^ explored the positive impact of the digital economy on industrial green total factor productivity based on data at the levels of China's industry as a whole and based on broad categories of industries, and they found that there was a threshold effect. By using provincial panel data, Hui et al.^19^ and Hao et al.^20^ found that the digital economy can effectively promote the high-quality development of the manufacturing industry. Ma et al.^21^ analyzed how the green total factor productivity of manufacturing industries in the Yellow River Basin was affected by the digital economy. Li et al.^22^ used a mediation effect model to demonstrate the causal mechanism between digital finance and corporate green innovation.

At present, the academic community has conducted a lot of research on the relationship between green total factors in manufacturing and the digital economy, but most of the research indicator systems are based on a regional perspective, often neglecting the measurement of the level of digital economic development in the manufacturing industry itself, which will reduce the credibility of the research conclusions to some extent. Some scholars use the financial reports of listed companies as the data source in their research on the industry. Although such research conclusions have high credibility, the data processing is complex, and many of the indicators are difficult to obtain and have a large number of missing values, which is not conducive to the extensive development of such research.

This study makes several important contributions to the literature.

1. This study builds a new indicator system to measure the level of digital economy development in the manufacturing industry from an industry perspective. Compared with existing research, this indicator system considers the three aspects of data production, data use, and industry informatization in the construction of the manufacturing industry, which is in line with the connotation and requirements of the development of the digital economy. This means that the indicator system in this study is more comprehensive and can complement the indicator systems constructed by other scholars. At the same time, compared with existing research, the data acquisition in this indicator system is easier and has few missing values, which can effectively reduce the amount of data processing in the study, thereby reducing the difficulty of the study.
2. Secondly, this study uses the Sobel mediation effect analysis method and Bootstrap final mediation effect analysis method with higher testing capabilities to clarify the mediating effect of the digital economy on the green total factor productivity of the manufacturing industry, and the conclusion is convincing. This can deepen our understanding of the role of the digital economy, which in turn is conducive to promoting the high-quality development of the manufacturing industry.
3. Finally, through the heterogeneity analysis of different types of manufacturing industries, this study draws a different conclusion from some studies: the development of the digital economy may have a certain degree of negative impact on the development of individual manufacturing industries. This conclusion will help the government and industry to formulate digital economy development strategies more cautiously.

The remainder of this paper is organized as follows. “Theoretical analysis and research assumptions” section describes the theoretical analysis conducted in this study and puts forward three hypotheses. “Research design” section presents the research model, the selection of the research indicators, and the specification of the data sources. “Analysis of empirical results” section describes an empirical analysis that was conducted with correlations and tests, and conducted a mediation effect analysis. “Further analysis: heterogeneity analysis” section describes a further heterogeneity analysis. “Discussion” section discusses the similarities and differences between this study and other existing studies. “Conclusions and policy implication” summarizes the conclusions of this research and makes relevant recommendations.

## Theoretical analysis and research assumptions

### Digital economy and green total factor productivity of the manufacturing industry

The core idea of Robert Solow's economic growth theory is that total factor productivity plays a decisive role in economic growth. Correspondingly, green total factor productivity also plays a decisive role in high-quality economic development. Therefore, this study takes green total factor productivity as an important research object with solid theoretical support. Further expanding Solow's economic growth theory, it can be concluded that technological progress is one of the important factors driving the improvement of green total factor productivity. Compared with traditional technological innovation, digital technology can more effectively improve production efficiency, reduce production inputs, and improve product quality, which also means that digital technology plays a key role in achieving the improvement of green total factor productivity. This conclusion has been verified to varying degrees in some scholars’ research^17–20^.

In addition, based on Schumpeter's innovation theory, technological progress will bring about institutional innovation, organizational innovation, and even changes in production methods, namely “disruptive innovation”. In fact, the emergence of digital technology has indeed brought about changes in economic development. Digital technology has promoted the development of the platform economy, which can shorten the time for transaction completion and reduce resource mismatch^17,23^; secondly, digital technology can help enterprises effectively collect and record various data generated during production and operation, which can provide a basis for relevant decision-making of enterprises and realize the transformation of enterprise operation mode, management mode, production mode, and improve financial performance^21,24^. At the same time, the digital economy can break down information barriers, break through traditional spatial and temporal limitations, and help various entities in economic activities quickly accumulate information and knowledge^25^, which can help enterprises with slower technological progress quickly narrow the technological gap with leading enterprises, achieve the improvement of the overall scientific and technological level of the industry, and further drive the improvement of the green total factor productivity of the industry^17^.

The digital economy is a direct product of the development and impact of digital technology, and will undoubtedly have a significant impact on green total factor productivity. Based on this, we propose the following hypothese:

#### H1

The digital economy can improve the overall green total factor productivity of the manufacturing industry.

### The digital economy affects green total factor productivity through a mediating effect

**Figure 1:**
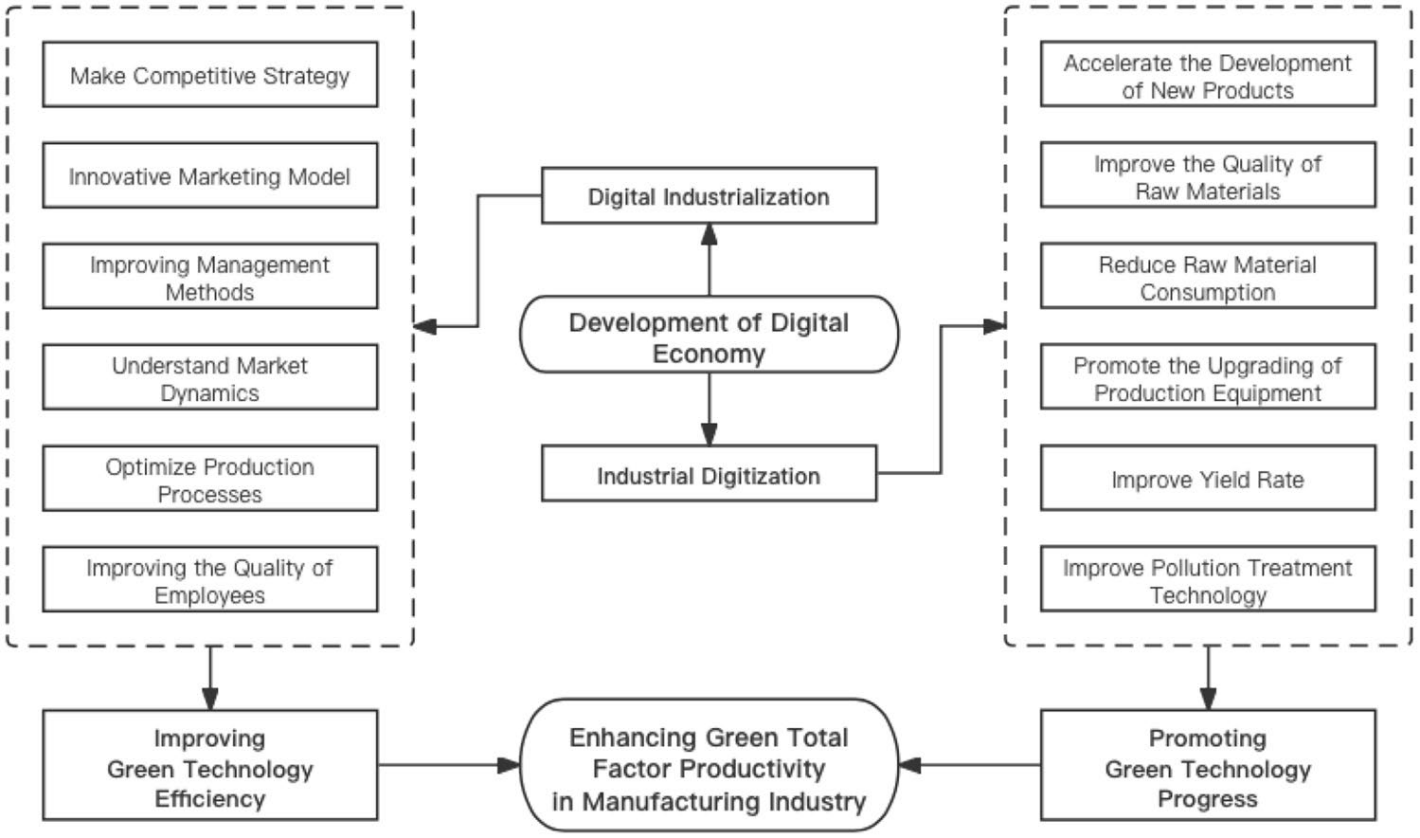
The mechanism of the impact of digital economy on GTFP of manufacturing industry.

Technological progress and technological efficiency progress are both important paths to promote changes in green total factor productivity (TFP). As mentioned in the previous section, the digital economy is generated with the development of digital technology. However, it must be noted that the vigorous development of the digital economy will have a counterproductive effect on the development of digital technology: mature digital technology will have the opportunity to be decentralized, promoted, and deepened, and traditional technology will also be able to deeply integrate with digital technology, thus achieving technological progress in society as a whole^8,18,19,26^. In addition, the digital economy also puts higher development requirements on various actors in the economic society, forcing changes in economic behaviors such as production, management, and training across the industry and even the whole society. These changes are reflected in economic models as changes in technological efficiency^8,26–28^. The mechanism of the impact of digital economy on GTFP of manufacturing industry can be shown in Fig. 1.

Based on this, we propose the following hypothese:

#### H2

The digital economy affects the green total factor productivity of the manufacturing industry by enhancing its technological progress or making changes in its technical efficiency.

### Different types of manufacturing industries have different mechanisms of response to the digital economy

The digital economy relies on the popularity of digital technology. However, due to the significant differences in production factors among different industries, this will affect the progress of the integration of various industries with digital technology. For example, some traditional industries rely on a large amount of labor or natural resources, while digital transformation involves more investment in technology, information, and capital. Therefore, the degree of digitalization of these traditional industries may be relatively low^28–30^. In theory, the higher the level of digitalization in an industry, the greater the dividends it can enjoy from the digital economy, and the more positive its impact on green total factor productivity. However, based on the perspective of externalities, some industries (often technology-intensive manufacturing industries) may face externalities in terms of digital transformation, such as data security, privacy protection, and other issues, which may make these industries less responsive to the digital economy than expected^30^. Based on the above situation, it is necessary to explore the impact of the digital economy on the green total factor productivity of different manufacturing industries in a classified manner. The lifecycle stages of different industries vary, and their technological innovation efficiency varies^31^, which also affects the degree of diffusion of the digital economy in the industry. Therefore, the following hypothese are proposed:

#### H3

There is heterogeneity in the impact of the development of the digital economy on the green total factor productivity of different categories of manufacturing industries.

## Research design

### Model setting

To test hypotheses H1 and H3, the following panel regression model was set up:

1 $$ gtfp_{i,t} = \beta_{0} + \beta_{1} ide_{i,t} + \theta X + \mu_{i} + \lambda_{t} + \epsilon_{i,t} $$

where $gtf{p}_{i,t}$ is the green total factor productivity in year $t$ for the broad category of industries $i$, $id{e}_{i,t}$ is the level of development of the digital economy in year $t$ for the broad category $i$, $X$ represents the control variables affecting green total factor productivity, ${\upbeta }_{0}$ is the intercept term, ${\upbeta }_{1}$ is the regression coefficient of the explanatory variables, $\mu$ is the fixed effect of the industry, $\lambda$ is the fixed effect of time, and $\epsilon$ is the residual term.

In order to test hypothesis H2, a mediating effect model will be established; the specific model setting and analysis methods will be described later.

### Variable construction, indicator selection, and data sources

#### Core explained variable

Green total factor productivity ($gtfp$), which considers pollution emissions (i.e., non-desired outputs) in the production process, can better evaluate the level of high-quality development of an industry's economy, and it is a green economy indicator that has attracted wide attention from academics in recent years. This study adopts the more mature super-efficient DEA-SBM model to measure it^19–21,32^.

##### DEA-SBM model

This model was proposed by Tone^33^ and can effectively solve the problem of indicator relaxation in traditional DEA models. This model assumes that there are $k$ decision-making units $DM{U}_{j}\left(j=\mathrm{1,2},\dots ,k\right)$, each DMU have m inputs and $n$ outputs. Among them, there are ${S}_{1}$ disired outputs (${y}^{g}$), ${S}_{2}$ non-disired outputs (${y}^{b}$). The DEA-SBM model formula for considering non-disired outputs is:

2 $$ \rho^{*} = min\frac{{1 - \frac{1}{m}\mathop \sum \nolimits_{i = 1}^{m} \frac{{S_{i}^{ - } }}{{x_{i0} }}}}{{1 + \frac{1}{{S_{1} + S_{2} }}\left( {\mathop \sum \nolimits_{r = 1}^{{S_{1} }} \frac{{S_{r}^{g} }}{{y_{r0}^{g} }} + \mathop \sum \nolimits_{r = 1}^{{S_{2} }} \frac{{S_{r}^{b} }}{{y_{r0}^{b} }}} \right)}} $$

$$ s.t.x_{0} = x\lambda - S^{ - } ,y_{0}^{g} = y^{g} \lambda - S^{g} ,y_{0}^{b} = y^{b} \lambda + S^{b} $$

$$ S^{ - } \ge 0,S^{g} \ge 0,S^{b} \ge 0,\lambda \ge 0 $$

Among them, $\rho$ is the efficiency evaluation index, ${S}^{-}$, ${S}^{g}$ and ${S}^{b}$ are relaxation variables, $\lambda$ is the weight of inputs and outputs indicator.

##### DEA-Malmquist index method

Fare et al.^34^ proposed the Malmquist productivity index to examine the growth of total factor productivity over two different periods, which can be used for multi input and multi output analysis based on panel data to reflect overall productivity changes. The expression for the variation of the malmquist index from $t$ to $t+1$ is:

3 $$ {\text{MI}}_{t}^{t + 1} = \left[ {\frac{{1 + D_{0}^{t} \left( {x^{t} ,y^{{g^{t} }} ,y^{{b^{t} }} ,S^{{*^{t} }} } \right)}}{{1 + D_{0}^{t} \left( {x^{t + 1} ,y^{{g^{t + 1} }} ,y^{{b^{t + 1} }} ,S^{{*^{t + 1} }} } \right)}} \times \frac{{1 + D_{0}^{t + 1} \left( {x^{t} ,y^{{g^{t} }} ,y^{{b^{t} }} ,S^{{*^{t} }} } \right)}}{{1 + D_{0}^{t + 1} \left( {x^{t + 1} ,y^{{g^{t + 1} }} ,y^{{b^{t + 1} }} ,S^{{*^{t + 1} }} } \right)}}} \right]^{\frac{1}{2}} $$

Among them, $x$ is the input factor, ${y}^{g}$ is the disired output, ${y}^{b}$ is the non-disired output, ${S}^{*}$ is the relaxation variable, and ${D}_{0}^{t}$ and ${D}_{0}^{t+1}$ is the distance function between the $t$ period and the $t+1$ period.

Furthermore, the MI index can be decomposed into changes in technological progress (TC) and changes in technological efficiency (EC):

4 $$ MI = TC \times EC $$

The MI index is the core explained variable in this paper: Green Total Factor Productivity (GTFP).

##### Index selection of DEA-SBM-Malmquist model

The physical capital stock, the size of the labor force, and the total energy consumption of the major types of manufacturing industries are taken as the input terms^8^; the added value of the industry is taken as the desired output term, and the COD and sulfur dioxide emissions are taken as the non-desired output terms^17^. The indicators for the DEA-SBM-Malmquist model can be shown in Table 1. For the physical capital stock, this study refers to the methods of Shan et al.^35^, Sun et al.^36^, and Yang^37^ and utilizes the amount and sequence of investments in the three types of fixed assets provided in the China Statistical Yearbook according to their compositions, followed by a calculation of the investment values of the three types of fixed-asset investments according to their compositions. Then, the depreciation of the three types of fixed-asset investments is calculated according to their compositions. Based on the different depreciation periods of different investment components, the corresponding depreciation rates under the condition of a geometrically decreasing relative efficiency are calculated; then, the capital stock of each component is calculated by using the perpetual inventory method and summed. All other indicators were obtained from the China Statistical Yearbook, China Industrial Statistical Yearbook, and China Environmental Statistical Yearbook.

**Table 1:** Selection of indicators for the DEA-SBM-Malmquist model.

Indicator type	Indicator name	Source of indicators	Indicator period
Inputs	The physical capital stock	Authors' own measurements	2010–2021
	The size of the labor force	China Statistical Yearbook and China Industrial Statistical Yearbook	
	The total energy consumption of the major types of manufacturing industries	China Statistical Yearbook and China Industrial Statistical Yearbook	
Desired Output	The added value of the industry	China Statistical Yearbook and China Industrial Statistical Yearbook	
Non-desired Output	Chemical oxygen demand (COD)	China Environmental Statistical Yearbook	
	Sulfur Dioxide Emissions	China Environmental Statistical Yearbook	

#### Core explanatory variables

The number of mobile phone subscribers, the number of mobile internet users, the length of fiber optic cables, the number of internet access ports, the revenue from software businesses, and investment in ICT were taken as the core explanatory variables. ICT investment and other indicators were used in the research and analysis. Focusing on the level of broad industrial categories, research on indicator systems for the level of development of the digital economy is relatively rare for two main reasons: First, according to the existing statistical surveys and public systems, most indicators (such as the number of computers in use at the end of a period, the number of websites owned by enterprises, etc.) are only published at the level of the sector in non-census years, and data with a smaller granularity can only be obtained from census yearbooks; such indicators have only been published since 2013, thus resulting in the problem of data disconnection. As a result, there is a data gap. Secondly, some important indicators that can reflect the level of digitization of an industry (such as the amount of software purchased, the amount of data services purchased, and computing power retained by enterprises, etc.) cannot be included in the existing statistical subjects due to a variety of reasons, which makes it impossible to build a more comprehensive system of indicators for evaluation. In summary, there are certain difficulties in measuring the level of development of the digital economy when using broad industrial data. However, after reading a large amount of the literature and looking through various statistical yearbooks, the authors of this study believe that it is still feasible to utilize the publicly available data at the level of broad categories in the yearbooks for the construction of an evaluation system for the level of digital economic development of the industry.

The core of the digital economy is the development and utilization of data elements through digital technology^38,39^. Therefore, the construction of the indicator system needs to consider the data production capacity and data usage capacity of the industry. Along with these capabilities, the industry should have certain information management capabilities^40^, as this is the basic requirement and intuitive manifestation of the industry's ability to carry out digital transformation. Therefore, the indicator system for the development level of the digital economy in the manufacturing industry in this study will be constructed from three aspects: data production capacity, data-use capacity, and information management capability.

In terms of data production capacity, drawing on the perspective of Zeng et al.^41^, the more R&D activities a company engages in, the more R&D data it generates. Therefore, it is reasonable to choose number of new products developed. The value of data elements in a company can be reflected in its financial indicators^42^, so operating expenses and total current assets can be used as metrics to measure the amount of production data generated by a company.

About data-use capabilities, firstly, data needs to be processed in computers, so the larger the number of computers in the industry, the stronger its data processing capabilities. Secondly, people, especially high-quality talents, are the core of analyzing and using data^42^, so industries with more highly educated talents also have stronger data utilization capabilities. Finally, the typical symbol of deep integration of the digital economy and manufacturing industry is to put data elements into the production process to realize digital production^39^, so the production equipment inventory of the industry is closely related to its data usage level. Therefore, this study chooses three indicators, namely, number of computers per 100 people, percentage of highly qualified personnel, and inventory of machinery and equipment, to measure the level of industry data-use capabilities.

In terms of level of information management, in order to adapt to the trends and requirements of the development of the digital economy, all industries in the manufacturing sector must raise the level of information technology transformation to a strategic level, and focus on strengthening the construction of industry management information systems, in order to gain advantages in market competition^43^. Therefore, when measuring the level of development of the digital economy in the industry, it is necessary to consider the level of information management in the industry. Therefore, it is reasonable for this study to include the indicators related to the level of information management in various industries provided in the China Economic Census Yearbook into the indicator system.

The construction of the specific index system is shown in Table 2.

**Table 2:** Index system for the evaluation of the level of development of the digital economy in an industry.

Primary indicators	Secondary indicators	Meaning of indicators
Data production capacity	Number of new products developed	Number of new product development activities in the industry; a larger number of developments means that the industry's scientific research activities are more abundant, which, in turn, will generate more R&D data
	Operating expenses	This includes selling expenses, management expenses, and financial expenses; more expenses mean more frequent business activities by an enterprise, thus allowing more abundant business data to be collected
	Total current assets	Total current assets can reflect the level of production activities in the industry; the higher the level of production activities, the greater the amount of valuable data generated
Data-use capabilities	Percentage of highly qualified personnel	Measured by the proportion of PhDs and masters in R&D organizations with respect to the total workforce; the more highly qualified personnel there are, the stronger the ability to integrate, analyze, and process data will be
	Inventory of machinery and equipment	The data measured in this study reflect the level of investment in machinery and equipment in the industry; the greater the inventory of machinery and equipment, the higher the level of computing power and the stronger the data processing capabilities
	Number of computers per 100 people	This reflects the level of computer penetration in the industry; the greater the number of computers per 100 people is, the higher the level of digitization in the industry will be
Level of information management	Share of enterprises with electronic financial management	Share of enterprises with informatized financial management; the greater the share, the more efficient the financial management, which, in turn, proves that the industry has a higher level of digitization
	Share of enterprises with electronic purchasing and inventory management	Share of enterprises that have realized the informatization of purchasing and inventory management; the rest of the explanation is the same as above
	Percentage of enterprises with computerized manufacturing management	Percentage of enterprises with computerized manufacturing management; the rest of the explanation is the same as above
	Share of enterprises with computerized logistical and distribution management	Share of enterprises with computerized logistical and distribution management; the rest of the explanation is the same as above
	Percentage of enterprises that have computerized their customer relationship management	Percentage of enterprises that have computerized customer relationship management; the rest of the explanation is the same as above
	Percentage of enterprises that have computerized human resource management	Percentage of enterprises that have implemented the informatization of human resource management; the rest of the explanation is the same as above

All data came from the China Statistical Yearbook, China Industrial Statistical Yearbook, China Science and Technology Statistical Yearbook, China Fixed-Asset Investment Statistical Yearbook, and China Economic Census Yearbook. For some missing data from non-census years, the data from the current census year were used as a substitute. Considering the different scales of the indicators, this study adopted the entropy value method, which is more objective, to assign weights to the above indicators and measure them collectively.

#### Selection of intermediary variables

According to formula (4), the GTFP index can be decomposed into Green Technology Progress (GTC) and Green Technology Efficiency Change (GEC). Green technology progress refers to the process in which enterprises achieve green product development, improve pollutant treatment capacity, reduce raw material consumption, and other green production methods by updating technology or equipment. It is an intuitive effect brought by industrial digitization. The efficiency change of green technology refers to the process in which enterprises achieve green development by optimizing organizational management, innovating marketing plans, and improving employee literacy, mainly relying on the development of digital industrialization. Both are important paths to improve green total factor productivity, but their focus is different. This paper will use these two indicators as intermediary variables to explore which path is more conducive to the improvement of green total factor productivity in the manufacturing industry.

The expression formulas for Green Technology Progress (GTC) and Green Technology Efficiency Change (GEC) are:

5 $$ {\text{GTC}}_{t}^{t + 1} = \left[ {\frac{{1 + D_{0}^{t + 1} \left( {x^{t} ,y^{{g^{t} }} ,y^{{b^{t} }} ,S^{{*^{t} }} } \right)}}{{1 + D_{0}^{t} \left( {x^{t} ,y^{{g^{t} }} ,y^{{b^{t} }} ,S^{{*^{t} }} } \right)}} \times \frac{{1 + D_{0}^{t + 1} \left( {x^{t + 1} ,y^{{g^{t} + 1}} ,y^{{b^{t} + 1}} ,S^{{*^{t + 1} }} } \right)}}{{1 + D_{0}^{t} \left( {x^{t + 1} ,y^{{g^{t + 1} }} ,y^{{b^{t + 1} }} ,S^{{*^{t + 1} }} } \right)}}} \right]^{\frac{1}{2}} $$

6 $$ {\text{GEC}}_{t}^{t + 1} = \frac{{1 + D_{0}^{t} \left( {x^{t} ,y^{{g^{t} }} ,y^{{b^{t} }} ,S^{{*^{t} }} } \right)}}{{1 + D_{0}^{t + 1} \left( {x^{t + 1} ,y^{{g^{t + 1} }} ,y^{{b^{t + 1} }} ,S^{{*^{t + 1} }} } \right)}} $$

The meaning of each indicator in both formulas is the same as in formula (4).

#### Other control variables

This study references existing relevant research and selects six control variables. Referring to the research of Shi and Sun^44^, the industry economic scale (lva) is represented by the logarithm of the industry value added in the current year. Referring to the research of Hu et al.^9^ and Wang^45^, the industry science and technology scale (lrd) is represented by the logarithm of the industry R&D expenditure in the current year. Referring to the research of Cheng and Qian^18^, the industry labor force scale (llab) is represented by the logarithm of the industry average labor force in the current year. Referring to the research of He et al.^46^ and Wang^47^, the industry pollution control fee (lptf) is represented by the logarithm of the industry pollution control facility operating cost in the current year. Referring to the research of Yang and Zeng^8^, the industry marketization level is measured by the proportion of state-owned main business income in total main business income. Referring to the research of Zeng et al.^41^ and Deng et al.^48^, the industry openness to the outside world (open) is measured by the proportion of foreign-funded enterprise main business income in total main business income.

#### Other explanations

Considering the changes in industrial classification standards during the sample period, in order to ensure the comparability of the sample, in line with the principle of keeping the data unchanged before and after their unification without splitting and while addressing past mergers, this study has adjusted and merged some of the categories in the manufacturing industry. The agricultural food processing and food manufacturing industries were merged into the food manufacturing industry, the textile, clothing, leather apparel, fur, feather and feather product, and footwear industries were merged into the textile, clothing, and fur product industry. The rubber and plastic manufacturing industries were merged into the rubber and plastic manufacturing industry. The general and special-purpose equipment manufacturing industries were merged into one category. The automobile, railway, shipbuilding, Aerospace, and other transportation equipment manufacturing industries were merged into the transportation equipment manufacturing category. For other manufacturing industries, such as the industry for the comprehensive utilization of waste resources, the metal product industry, and the machinery and equipment repair industry, because the initial year of statistics was relatively late and multiple changes in the industry segmentation, the amounts of missing data were relatively high. Therefore, these three industries were excluded from this study to ensure the reliability of the conclusions. After the adjustments and necessary modifications made in the above steps, this study finally regrouped China's manufacturing industries into 23 major categories, as shown in Table 3.

**Table 3:** Adjusted broad classification of manufacturing industries.

No	Industry name	No	Industry name
T01	Food Manufacturing	T13	Chemical Fiber Manufacturing
T02	Beverage Manufacturing	T14	Rubber and Plastic Product Industry
T03	Tobacco Product Industry	T15	Non-metallic Mineral Product Industry
T04	Textile, Clothing, and Fur Product Industry	T16	Ferrous Metal Smelting and Calendaring Industry
T05	Wood Processing and Wood, Bamboo, Rattan, Palm, and Grass Products	T17	Nonferrous Metal Smelting and Rolling Processing Industry
T06	Furniture Manufacturing	T18	Metal Product Industry
T07	Paper and Paper Product Industry	T19	General and Special-Purpose Machinery Manufacturing Industry
T08	Printing and Recording Media Reproduction	T20	Transportation Equipment Manufacturing
T09	Cultural, educational, industrial and aesthetic, sports, and recreational supply manufacturing	T21	Electrical machinery and equipment manufacturing
T10	Petroleum, coal, and other fuel processing industry	T22	Computer, communication, and other electronic equipment manufacturing industry
T11	Chemical raw material and chemical product manufacturing industry	T23	Instrument and meter manufacturing industry
T12	Pharmaceutical Manufacturing		

In order to ensure that the data were comparable across periods, all of the above indicators involving prices were converted according to the corresponding price indices, with the 1990 prices as the basis. Considering the data availability, this study selected the industrial data described above for analysis. Further, considering that the standards of these industries were adjusted from a main business income of 5 million and above to 20 million and above in 2011, in order to prevent sample bias from affecting the conclusions of the analysis, a time span from 2011 to 2021 was chosen for each indicator. For some of the missing values, the average annual growth rate was utilized in the calculations. Table 4 shows the definition of each variable and the results of the descriptive statistics.

**Table 4:** Descriptive statistics of the variables.

Variable	Variable definition	Sample size	Mean	Standard deviation	Minimum	Maximum
gtfp	Green total factor productivity	253	1.444	0.713	0.589	4.543
ide	Level of development of the digital economy in the industry	253	0.255	0.166	0.0150	0.871
lva	Size of the industrial economy	253	7.503	0.874	5.419	8.971
lrd	Industry technology size	253	4.376	1.275	1.203	6.889
llab	Industry labor force size	253	5.401	1.007	2.754	7.188
lptf	Industry pollution control costs	253	11.26	1.761	7.062	14.62
market	Degree of marketization in the industry	253	0.183	0.227	0.00686	0.997
open	Degree of openness of the industry	253	0.223	0.125	0	0.757

## Analysis of empirical results

### Analysis of benchmark regression results

Table 5 shows the results of the benchmark regression of the impact of the level development of the digital economy on each industry's green total factor productivity. Model (1) did not include control variables, and models (2) to (6) included control variables; models (2), (4), and (6) were all fixed-effect models, and models (3) and (5) were both random-effect models. Models (2) and (3) involved industry effect fixation, models (4) and (5) involved time effect fixation, and model (6) involved a double fixation of the industry effect and time effect. The coefficients of the core explanatory variables from model (1) to model (6) were all significantly positive, indicating that the development of the digital economy had a significant positive effect on the green total factor productivity in the manufacturing industry, thus confirming hypothesis H1 of this study.

**Table 5:** Baseline regression results.

	(1)	(2)	(3)	(4)	(5)	(6)
	gtfp	gtfp	gtfp	gtfp	gtfp	gtfp
ide	2.282***	3.155***	3.019***	1.016**	2.149***	2.467***
	(0.627)	(0.614)	(0.552)	(0.501)	(0.563)	(0.608)
lva	–	0.579**	0.631***	− 0.252	0.615***	0.746***
		(0.228)	(0.165)	(0.166)	(0.149)	(0.259)
lrd	–	0.113	− 0.090	− 0.048	− 0.086	0.370**
		(0.159)	(0.117)	(0.082)	(0.092)	(0.161)
llab	–	− 0.702***	− 0.986***	0.156	− 0.790***	− 0.509***
		(0.147)	(0.103)	(0.151)	(0.116)	(0.184)
lptf	–	0.206***	0.172***	− 0.023	0.006	0.144***
		(0.043)	(0.041)	(0.037)	(0.041)	(0.044)
market	–	1.470	− 2.129***	0.321	− 2.043***	− 1.107
		(1.119)	(0.465)	(0.382)	(0.312)	(1.134)
open	–	1.142**	0.259	− 0.949**	− 0.322	0.658
		(0.542)	(0.476)	(0.377)	(0.434)	(0.509)
_cons	0.574***	− 3.255**	0.057	2.856***	1.307**	− 4.876***
	(0.130)	(1.581)	(0.917)	(0.477)	(0.521)	(1.670)
N	253	253	253	253	253	253
R^2^	0.669	0.651	0.220	0.066	0.281	0.724
Id FE	Yes	Yes	Yes	No	No	Yes
Year FE	Yes	No	No	Yes	Yes	Yes

***, **, and * denote significance levels of 1%, 5%, and 10%, respectively; robust standard errors are in parentheses. The symbols have the same meaning below.

Further analysis of other control variables:

The coefficient of the industry economic scale index (lva) was significantly positive in most models, indicating that the expansion of an industry’s scale can allow the scale effect to be realized, which is conducive to the intensive development of that industry; thus, this plays a positive role in improving green total factor productivity. However, the coefficient of the industry labor force scale indicator (llab) was significantly negative in most models, implying that an increase in labor force would have a negative impact on green total factor productivity, which was probably because a large labor force input tends to hinder the popularization of digitalization and mechanization in an industry, reduce labor productivity, and increase the additional loss and waste in the production process, resulting in a decrease in the overall green total factor productivity.

### Robustness test

#### Adjusting the sample period

1. The COVID-19 pandemic had a large impact on China's economic development in 2020 and 2021, so these years were excluded. However, at the same time, the emergence of new behaviors, such as big data trip tracking, big data streaming, telecommuting, and online lectures, also objectively promoted the development of China's digital economy. Therefore, the relevant data in this period may have had an abnormal impact on the regression results, so the data from these years were excluded before the regression analysis, and the results are shown in column (1) of Table 6. The results show that after the exclusion of the possibly abnormal impact of the COVID-19 pandemic, the positive effect of the digital economy on the green total factor productivity was still robust.
2. Exclusion of the year of the impact of the digital economy policy: In 2017, the digital economy was written into a government work report for the first time, and since then, the development of China's digital economy has been on a fast track. In order to exclude the possible impact of this policy, the sample period was shortened to 2011–2016, and the regression results are shown in Table 6, column (2). The coefficients of the core explanatory variables were found to be significant, proving that the conclusions were still robust.

**Table 6:** Robustness test.

	(1)	(2)	(3)	(4)	(5)	(6)	(7)
	gtfp	gtfp	gtfp	gtfp	gtfp	gtfp	gtfp
ide	2.068***	2.091***	2.473***			2.549***	2.624***
	(0.673)	(0.728)	(0.774)			(0.604)	(0.598)
cride				2.031**	2.946***		
				(0.870)	(0.846)		
lva	0.909***	0.923***	0.716**		0.833***	0.781***	0.538**
	(0.215)	(0.168)	(0.281)		(0.259)	(0.262)	(0.261)
lrd	0.078	− 0.061	0.417**		0.348**	0.411**	0.516***
	(0.154)	(0.135)	(0.172)		(0.162)	(0.165)	(0.174)
llab	− 0.390***	− 0.325***	− 0.495**		− 0.475**	− 0.533***	− 0.624***
	(0.145)	(0.111)	(0.195)		(0.185)	(0.184)	(0.174)
lptf	0.085*	0.067*	0.146***		0.149***	0.143***	0.162***
	(0.044)	(0.036)	(0.046)		(0.044)	(0.043)	(0.045)
market	− 1.647	− 2.219**	− 1.187		− 1.515	− 1.048	− 0.540
	(1.012)	(0.923)	(1.199)		(1.143)	(1.131)	(1.145)
open	0.363	0.180	1.530		0.057	0.641	0.982
	(0.509)	(0.701)	(1.054)		(0.523)	(0.512)	(0.652)
_cons	− 4.681***	− 4.259***	− 5.102***	0.384	− 5.832***	− 5.170***	− 3.839**
	(1.418)	(1.157)	(1.779)	(0.276)	(1.677)	(1.705)	(1.856)
N	207	161	231	253	253	253	253
R^2^	0.644	0.645	0.716	0.657	0.719	0.726	0.726
Id FE	Yes	Yes	Yes	Yes	Yes	Yes	Yes
Year FE	Yes	Yes	Yes	Yes	Yes	Yes	Yes

Due to space constraints, the coefficients and significance of the control variables are not shown, but they can be obtained by contacting the author if needed.

*Note*: ***, **, * denote significant at the 1%, 5% and 10% levels respectively.

#### Adjusting the sample size

Referring to the practice of Shi et al.^44^, the relevant data from the computer, communication, and other electronic equipment manufacturing industry were excluded. At the same time, it was found that the tobacco industry is a special industry that is highly controlled by the state, and its indicators were quite different from those of other industries, so its relevant data were also excluded. The regression results are shown in Table 6, column (3); it was found that the core explanatory variables’ coefficients were significantly positive, proving that the conclusion was still robust.

#### Replacing measurement method for the core explanatory variables

This study utilized the CRITIC weighting method to measure the level of development of the digital economy of the industries again. The CRITIC weighting method is also an objective weighting method; it is based on volatility and conflicts in the data to determine the weight of each indicator, and it is often used in datasets where there are correlations among the indicators. The data of the core explanatory variables in this study were reevaluated with this method to obtain a proxy variable (CRIDE) for the original level of digital economic development. The results of a regression after its substitution into the model are shown in Table 6, columns (4) and column (5). Column (4) does not include control variables, and column (5) includes control variables. The results show that the coefficients were significantly positive, thus proving the robustness of the previous findings.

#### Shrinking the control variables

This study did not shrink the control variables in the baseline regression model, but in the process of data organization, it was found that there were extreme values in the control variables. Therefore, bilateral shrinkage of the control variables at the 1% and 5% levels was performed to reduce the impact of extreme values. The corresponding regression results are shown in column (6) and column (7) of Table 6, respectively. The results show that the coefficients of the core explanatory variables were significantly positive at the 1% level, which was consistent with the previous findings, thus further proving the robustness of the findings of this study.

### Endogeneity test

The model in this study had an endogeneity problem: On the one hand, variables in the model had to be omitted due to the availability of statistical data; on the other hand, there was a mutual causal relationship between the digital economy and the green total factor productivity because industries with higher green total factor productivity tend to pay more attention to the application of new technologies, i.e., they are more incentivized to invest in the construction of the digital economy. In order to make the conclusions more robust and reliable, this study adopted the fixed-effect method and instrumental variable method to explore the issue of endogeneity.

#### Fixed-effect method

This study adopted a double fixed-effect model that took the differences between years and industries into account for the benchmark regression (6), and the regression results were significant at the 1% level, proving that the model mitigated the endogeneity problem caused by the omission of variables to a certain extent.

#### Instrumental variable method

Two instrumental variables were constructed in order to better mitigate the endogeneity problem. Referring to Chen et al.^49^, the one lagged period of the core explanatory variables was used as the first instrumental variable; drawing on the ideas of Nunn et al.^50^, Huang et al.^51^, and Guo et al.^10^, the percentage of enterprises in the major categories of manufacturing industries using LANs in 2013 was selected for comparison with that percentage in previous years. The second instrumental variable was constructed by multiplying the number of patent applications in each industry category in 2013. After that, the endogeneity problem was tested with the 2SLS method and system GMM method, and the results are shown in Table 7. Columns (1)-(3) show the results of the 2SLS method, and columns (4)-(6) show the results of the system GMM method. Columns (1) and (4) involved the input of only the first instrumental variable, columns (2) and (5) involved the input of only the second instrumental variable, and columns (3) and (6) involved the input of the two instrumental variables.

**Table 7:** Endogeneity test.

	(1)	(2)	(3)	(4)	(5)	(6)
	gtfp	gtfp	gtfp	gtfp	gtfp	gtfp
ide	2.542***	2.904***	2.835***	2.942*	3.235**	2.935**
	(0.950)	(0.532)	(0.884)	(1.637)	(1.506)	(1.356)
lva	1.268*	0.714***	1.172*	0.430	0.367	0.512
	(0.709)	(0.238)	(0.703)	(0.458)	(0.352)	(0.406)
lrd	0.599***	0.383**	0.605***	− 0.101	− 0.179	− 0.134
	(0.202)	(0.158)	(0.203)	(0.286)	(0.214)	(0.213)
llab	− 1.173***	− 0.526***	− 1.195***	− 0.764***	− 0.625**	− 0.770***
	(0.440)	(0.155)	(0.444)	(0.286)	(0.269)	(0.290)
lptf	0.126**	0.148**	0.129**	− 0.008	0.008	− 0.009
	(0.064)	(0.060)	(0.065)	(0.091)	(0.091)	(0.102)
market	− 0.753	− 1.060	− 0.709	− 1.286	− 1.733	− 2.068**
	(0.937)	(0.858)	(0.926)	(2.502)	(1.428)	(1.006)
open	0.967*	0.696	0.962*	− 0.927	− 0.681	− 0.469
	(0.579)	(0.495)	(0.580)	(1.742)	(0.981)	(0.832)
_cons	− 6.816	− 5.681***	− 6.035	2.516	2.349	2.133
	(5.223)	(2.031)	(5.238)	(1.649)	(1.476)	(1.601)
N	230	253	230	230	253	230
r2	0.815	0.803	0.815	–	–	–
Id FE	Yes	Yes	Yes	Yes	Yes	Yes
Year FE	Yes	Yes	Yes	Yes	Yes	Yes
Kleibergen-Paap rk LM statistic	28.576***	26.061***	29.051***	–	–	–
Kleibergen-Paap rk Wald F statistic	228.900 [16.38]	605.827 [16.38]	302.428 [19.93]	–	–	–

*Note*: ***, **, * denote significant at the 1%, 5% and 10% levels respectively.

The Kleibergen-Paap rk LM statistics shown in column (1) and column (2) of Table 7 were significant at the 1% level, and the Kleibergen-Paap rk Wald F statistic was greater than 10, implying that there was no problem of unidentifiable instrumental variables or weak instrumental variables, which suggested that the above two instrumental variables were valid. The coefficients of the core explanatory variables shown in the columns of Table 7 were significantly positive, which was consistent with the findings in the benchmark regression, proving that the conclusions of this study are still robust after considering the endogeneity issue.

### Mediating effect analysis

Green total factor productivity can be decomposed into green technical efficiency change (GEC) and green technical progress change (GTC). To further analyze the effects of the digital economy on green total factor productivity, a mediating effect model was established.

Currently, a three-stage model is commonly used in the literature to analyze mediating effects, and such a model is constructed as follows:

7 $$gtf{p}_{i,t}={\beta }_{0}+=c*id{e}_{i,t}+\theta X+{\mu }_{i}+{\lambda }_{t}+{\epsilon }_{i,t}$$

8 $$gt{c}_{i,t}={\upbeta }_{0}+{a}_{1}id{e}_{i,t}+\theta X+{\upmu }_{i}+{\uplambda }_{t}+{\upepsilon }_{i,t}$$

9 $$gt{fp}_{i,t}={\upbeta }_{0}+{c}_{1}{\prime}id{e}_{i,t}+{b}_{1}gt{c}_{i,t}+\theta X+{\upmu }_{i}+{\uplambda }_{t}+{\upepsilon }_{i,t}$$

10 $$gtf{p}_{i,t}={\beta }_{0}+=c*id{e}_{i,t}+\theta X+{\mu }_{i}+{\lambda }_{t}+{\epsilon }_{i,t}$$

11 $$ge{c}_{i,t}={\beta }_{0}+{a}_{2}id{e}_{i,t}+\theta X+{\mu }_{i}+{\lambda }_{t}+{\epsilon }_{i,t}$$

12 $$gt{fp}_{i,t}={\beta }_{0}+{c}_{2{\prime}}id{e}_{i,t}+{b}_{2}ge{c}_{i,t}+\theta X+{\mu }_{i}+{\lambda }_{t}+{\epsilon }_{i,t}$$

where $gtc$ is the change in technical progress and $gec$ is the change in technical efficiency; these are the mediating variables used in this study. The coefficient $c$ is the total effect, the coefficients ${a}_{1}$ and ${a}_{2}$ are the effects of the core explanatory variables on the mediating variables, the coefficients ${b}_{1}$ and ${b}_{2}$ are the effects of the mediating variables on the explained variables after controlling for the effects of the core explanatory variables, and the coefficients $c_{1}^{\prime }$ and $c_{2}^{\prime }$ are the direct effects of the explanatory variables on the explained variables after controlling for the effects of the mediating variables. The meanings of the remaining variables are the same as those in Eq. (1) in the previous section.

The model’s results are shown in Table 8. According to the results of the sequential test method, the coefficient of $ide$ on $gtfp$ ($c$) was significant at the 1% level; the coefficient of $ide$ on $gtc$ (${a}_{1}$) was significant at the 1% level; the coefficient of $gtc$ (${b}_{1}$) was significant at the 1% level after the simultaneous application of $ide$ and $gtc$, while the total effect ($c_{1}^{\prime }$) was not significant. The coefficient of ide on $gec$ (${a}_{2}$) was not significant; the coefficient of ide on $gec$ (${b}_{2}$) was significant at the 1% level, and the total effect ($c_{2}^{\prime }$) was significant at the 1% level after the simultaneous application of $ide$ and $gec$. The three-stage mediating effect model required all coefficients to be significant in order to conclude that the mediating effect was established. The results showed that the coefficients were not significant, meaning that $gtc$ and $gec$ did not pass the mediating effect test, and the mediating effect was not established.

**Table 8:** Results of the first three-stage mediating effect test.

	(1)	(2)	(3)	(4)	(5)
	gtfp	gtc	gtfp	gec	gtfp
ide	2.467***	4.370***	0.011	− 0.875	2.853***
	(0.608)	(0.467)	(0.652)	(0.574)	(0.557)
gtc			0.562***		
			(0.081)		
gec					0.441***
					(0.066)
lva	0.746***	− 0.034	0.723***	0.765***	0.427*
	(0.259)	(0.199)	(0.244)	(0.234)	(0.241)
lrd	0.370**	0.409***	− 0.039	0.141	0.388***
	(0.161)	(0.124)	(0.152)	(0.149)	(0.146)
llab	− 0.509***	− 0.119	− 0.334*	− 0.442***	− 0.362**
	(0.184)	(0.141)	(0.174)	(0.167)	(0.169)
lptf	0.144***	0.152***	− 0.000	0.059	0.144***
	(0.044)	(0.034)	(0.041)	(0.041)	(0.040)
market	− 1.107	− 0.787	− 0.053	− 0.664	− 1.084
	(1.134)	(0.872)	(1.071)	(1.027)	(1.033)
open	0.658	0.270	0.427	0.507	0.470
	(0.509)	(0.391)	(0.481)	(0.460)	(0.465)
_cons	− 4.876***	− 2.014	− 3.743**	− 2.086	− 3.956**
	(1.670)	(1.285)	(1.519)	(1.578)	(1.528)
N	253	253	253	253	253
R^2^	0.724	0.769	0.776	0.317	0.772
Id FE	Yes	Yes	Yes	Yes	Yes
Year FE	Yes	Yes	Yes	Yes	Yes

*Note*: ***, **, * denote significant at the 1%, 5% and 10% levels respectively.

However, some scholars have clearly pointed out that the testing power of a sequential test in a three-stage mediating effect model is the lowest^52^ because, even if the sequential test is not passed, this does not mean that the mediating effect is not established. In order to avoid bias, this study then used the Sobel method and bootstrap method to test whether the mediating effect described in the hypotheses was established. The test results are shown in Tables 9 and 10.

**Table 9:** Results of the mediating effect test with Sobel's method.

Content of the test	ide → gtc → gtfp			ide → gec → gtfp		
	Coef	Std Err	P >|Z|	Coef	Std Err	P >|Z|
ax	4.025***	0.493	0.000	− 0.731	0.553	0.186
bx	0.649***	0.074	0.000	0.472***	0.070	0.000
cx	2.282***	0.627	0.000	2.282***	0.627	0.000
abx	2.613***	0.437	0.000	− 0.345	0.266	0.195
c'x	− 0.331	0.617	0.591	2.627	0.573	0.000
sobel	2.613	0.437	0.000	− 0.345	0.266	0.195
goodman-1	2.613	0.439	0.000	− 0.345	0.269	0.199
goodman-2	2.613	0.436	0.000	− 0.345	0.263	0.190
ab/c'(|ab/c'|)	− 7.885 (7.885)			− 0.132 (0.132)		

*Note*: ***, **, * denote significant at the 1%, 5% and 10% levels respectively.

**Table 10:** Results of the mediating effect test with the bootstrap method.

	ide > gtc > gtfp		ide > gec > gtfp	
	ind_eff	dir_eff	ind_eff	dir_eff
Observed coefficient	2.456*** (0.519)	0.011 (0.786)	− 0.386 (0.266)	2.853*** (0.630)
z	4.73	0.01	− 1.45	4.53
P >|z|	0.000	0.989	0.147	0.000
Normal-based [95% conf. interval]	[1.439–3.474]	[− 1.52–1.551]	[− 0.907, 0.136]	[1.617, 4.088]
0 in 95% conf	No	Yes	Yes	No
Reps	1000		1000	
CVs	Yes		Yes	
Id FE	Yes		Yes	
Year FE	YEs		Yes	
Eff_type	Indirect		Direct	

According to the results of the Sobel method, the coefficients of the mediating variable $gtc$ were all significant at the 1% level in the Sobel test, Goodman1 test, and Goodman2 test, and they passed the mediation test, so the mediating effect was considered to be established. The mediating variable $gec$ failed the above test, and its mediating effect was not considered to be established.

The bootstrap method assumes that if the confidence interval of an effect does not contain 0, the effect can be recognized as valid, and vice versa. After 500 random samples, the confidence interval of the mediating variable $gtc$ for the mediating effect did not contain 0, while the confidence interval of the direct effect contained 0; so, it could be hypothesized that there was a mediating effect and that there was no direct effect. The confidence interval of the direct effect of the mediating variable $gec$ did not contain 0, while the confidence interval of the mediating effect contained 0, so it could be hypothesized that there was a direct effect and that there was no mediating effect.

The digital economy cannot be directly inserted into production like other physical factors of production, but it can empower the existing production behavior of the manufacturing industry by promoting the upgrading and innovation of manufacturing equipment, improving the overall level of science, technology, and digitization of the industry, generally improving the production capacity of the manufacturing industry, reducing the energy consumption and emissions, and allowing improvement in the overall green total factor productivity of the manufacturing industry to be realized. However, this model showed that this improvement was not obvious ($c_{1}^{\prime }$ was not significant), which was presumed to be due to the fact that, on the one hand, the industry's investment in the digital transformation of green production technology was still low and the enthusiasm was not high; on the other hand, it was because the digital economy's empowerment of green production technology had not yet been deepened^53^, which led to an increase in the capacity for pollutant treatment.

In addition, the energy rebound effect caused by digital economy technologies may also have a negative impact on energy saving and emission reduction, but this needs to be further verified and analyzed. However, it should be clear that the digital economy has significantly improved the industry's green technological progress^53,54^ (the coefficient ${a}_{1}$ was significantly positive), so if the industry's GTFP is to be rapidly improved in the short term, it is important to increase the investment in green technological progress in a timely manner.

The three tests suggest that the digital economy does not bring about changes in green total factor productivity through changes in technical efficiency; however, this should not lead to the hasty conclusion that technical efficiency fails to play a role in it, and this requires further analysis. The regression results in Table 8 show that the coefficient ${a}_{2}$ was insignificant and negative in sign, and the coefficients ${b}_{2}$ and $c_{2}^{\prime }$ were significant and positive in sign. Referring to the interpretations of Wen et al. ^52^ and Liu et al.^55^, it was found that $a*b$ was heteroscedastic with the sign of $c_{2}^{\prime }$, and an argument should be made for the masking effect. There is a masking effect of changes in technical efficiency on green total factor productivity, i.e., at this stage, the digital economy enhances the industry's green total factor productivity by reducing its green technical efficiency. This finding does not prove that the digital economy does not have a positive effect on green technical efficiency, but it reflects the fact that the current factors affecting green technical efficiency, such as industry organization and management, environmental awareness, and workforce quality, do not match the reality of the rapid development of the digital economy. This is, on the one hand, because the digital economy has had an impact on the traditional organization of production and management behavior. On the other hand, this also shows that industry practitioners have failed to improve their own awareness of and literacy in the digital economy in a timely manner^56^, and these aspects of the improvement and enhancement of the industry often need to last for a certain period of time; that is, there will be lagging factors. The specific impacts also need to be further explored with the development of society. It should be noted that the coefficients ${b}_{2}$ and $c_{2}^{\prime }$ in the model were significantly positive, which precisely proved that green technology efficiency is an important way to enhance green total factor productivity^57^, and this must receive the industry's long-term attention.

## Further analysis: heterogeneity analysis

The manufacturing industry is one of China's main industries, and it has many classifications and various characteristics. There are differences in the responsiveness of different industries to the digital economy. In order to verify hypothesis H3, a group regression was carried out on 23 manufacturing industries to conduct a heterogeneity analysis.

According to the results of the benchmark regression, labor inputs had a negative impact on green total factor productivity, and it is speculated in this study that the digital economy may have a special impact on labor-intensive industries, so labor-intensive manufacturing industries were taken as the first subgroup. Green total factor productivity considers energy consumption and pollution emissions in addition to conventional input–output items, so highly polluting, energy-intensive, and water-intensive industries are the focus of attention. In this study, industries with total pollution emissions that were higher than the average emission level of the manufacturing industry were classified as high-pollution industries and were classified into the second subgroup. Some industries (such as the food manufacturing and textile industries) that are labor-intensive in the traditional sense were included in this subgroup because their pollution emissions were much higher than the average level of the manufacturing industry. The remaining industries were all technology-intensive manufacturing industries, and they were categorized into the third subgroup. The grouping results are shown in Table 11.

**Table 11:** Criteria for the division of manufacturing industries into broad categories.

Manufacturing grouping criteria	Included broad industry categories
Labor-intensive manufacturing	T02 T03 T05 T06 T08 T09 T12 T13 T14
Highly Polluting Manufacturing	T01 T04 T07 T10 T11 T15 T16 T17
Technology-intensive manufacturing	T18 T19 T20 T21 T22 T23

The results of the grouping regression are shown in Table 12. Columns (1), (3), and (5) in Table 12 correspond to the results of the grouping regression without control variables, and columns (2), (4), and (6) present the results of the grouping regression with control variables.

**Table 12:** Regression results for the large groupings of manufacturing industries.

Division	Labor-intensive manufacturing		Highly polluting manufacturing		Technology-intensive manufacturing	
	(1)	(2)	(3)	(4)	(5)	(6)
	gtfp	gtfp	gtfp	gtfp	gtfp	gtfp
ide	− 6.846***	− 9.650***	6.458***	7.422***	0.473	− 0.506
	(2.372)	(2.278)	(2.028)	(2.624)	(0.984)	(1.111)
lva		1.082**		1.599***		1.299*
		(0.475)		(0.364)		(0.699)
lrd		0.122		0.347		− 2.007**
		(0.254)		(0.256)		(0.829)
llab		− 0.746***		− 0.538**		− 0.196
		(0.272)		(0.259)		(0.590)
lptf		0.082		− 0.072		− 0.044
		(0.101)		(0.220)		(0.054)
market		− 10.833***		− 1.359		3.594
		(2.444)		(1.523)		(3.452)
open		− 0.485		2.804*		0.663
		(0.704)		(1.480)		(0.937)
_cons	1.848***	− 0.189	− 0.067	− 9.895***	0.854**	2.203
	(0.323)	(2.932)	(0.350)	(3.558)	(0.333)	(4.099)
N	99	99	88	88	66	66
R^2^	0.660	0.797	0.630	0.752	0.862	0.902
id FE	Yes	Yes	Yes	Yes	Yes	Yes
year FE	Yes	Yes	Yes	Yes	Yes	Yes

*Note*: ***, **, * denote significant at the 1%, 5% and 10% levels respectively.

### Labor-intensive manufacturing industries

The coefficients of the core explanatory variables in columns (1) and (2) of Table 12 were significantly negative at the 1% level, indicating that the digital economy had a significant negative impact on the green total factor productivity of labor-intensive manufacturing industries. There were three main reasons for this.

First, labor itself is irreplaceable. Human intelligence is irreplaceable, at least under the current technological conditions; product design and the personalization required on the market cannot be completely performed by artificial intelligence, as human intelligence is required to identify and meet such requirements. Secondly, human operations cannot be replaced. Although some repetitive production processes can be taken over by machines, certain production processes, such as engraving, cutting, and other fine craft operations, as well as the production experience that is summarized in practice and other factors that cannot be quantified, cannot be taken over by means of digital production^58,59^. Therefore, if such industries are blindly digitized, they tend to reduce their production efficiency, increase their material inputs, and increase their waste emissions.

Second, the development of the digital economy requires a large amount of capital and technological investment, which is a shortcoming of labor-intensive manufacturing industries. The irreplaceability of labor means that labor-intensive enterprises have to pay much in wages, which reduces their investments in technological innovation and equipment acquisition and, in turn, leads to a slow growth of or even decrease in their green productivity.

Third, the blind intervention of the digital economy has an impact on employment in labor-intensive enterprises^60–62^. On the one hand, the rapid development of the digital economy will shift more jobs to non-labor-intensive industries, which will lead to a serious labor shortage, leading to the reduction of green productivity. On the other hand, since some low-end jobs may be replaced by digital production and the labor quality of the replaced labor force often cannot support their competence in other jobs, this will bring about social problems, such as an increase in the unemployment rate; Thus, labor-intensive industries are relatively less enthusiastic about the introduction of the digital economy, which also indirectly leads to a reduction in their green total factor productivity^58,59,63,64^.

### Highly polluting manufacturing industries

The coefficients of the core explanatory variables in columns (3) and (4) in Table 12 were significantly positive at the 1% level, indicating that the development of the digital economy had a positive effect on the improvement of green total factor productivity in highly polluting manufacturing industries. There were three reasons for this.

First, the absolute amounts of energy and raw materials invested and pollutants emitted in highly polluting industries are large. The digital economy can enhance the output efficiency of such industries’ various production links to achieve a substantial reduction in the units output with respect to the input; the digital economy can also promote such industries’ innovation of green production technology so that their production processes and procedures are more in line with environmental protection requirements, thus reducing the pollution of the various production links to make it easier to achieve the purpose of emission reduction. The direct manifestation of this is that the green total factor productivity of high-pollution industries has experienced a substantial increase due to the intervention of the digital economy^65–67^.

Second, the technology involved in the digital economy can enhance the pollution treatment efficiency of high-pollution industries, which, on the one hand, increases their pollutant treatment capacity and reduces the emission of non-compliant pollutants; on the other hand, it can reduce the unit cost of pollutant treatment and lower such industries’ investment in pollution treatment, making them more willing and able to carry out pollution treatment activities.

Thirdly, high-pollution industries are the focus of the environmental protection department in the context of China's high-quality economic development. The digital economy can effectively improve the abilities of high-pollution industries to identify policy risks, access the demand for green products in the market, and quickly dominate market competition, which can allow them to transform green production, pollution control, and other practices that they originally needed to passively follow into proactive behaviors that enterprises are willing to carry out in order to actively cope with changes in the market, reduce the difficulty of implementing energy-saving and emission reduction policies, and, thus, improve their green total factor productivity^68,69^.

### Technology-intensive manufacturing

The coefficients of the core explanatory variables in columns (5) and (6) of Table 12 were not significant at the 10% level, and the signs of the coefficients changed after the application of the control variables, indicating that the increase in the level of the digital economy had a very limited impact on the green total factor productivity of technology-intensive manufacturing industries. The reasons for this were more complex.

First, due to the characteristics of technology-intensive industries themselves, the absolute amount of their pollutant emissions is small; thus, the effect of the development of the digital economy on pollutant emissions is limited; to some extent, the digital economy can even lead to an increase in the pollutant emissions of technology-intensive enterprises^68,70^.

Second, the development of the digital economy in technology-intensive industries started earlier, and the improvements in productivity, reductions in raw material consumption, and reductions in pollutant emission brought about by the popularization of various digital technologies have already reached a certain level, so the further intervention of the digital economy did not have a significant effect on their green total factor productivity.

Third, unlike highly polluting industries that pay more attention to energy-saving and emission reduction activities to avoid penalties, technology-intensive industries pay more attention to R&D for new technologies and new iterations of products than to reductions in pollutant emissions and energy consumption, which shows that such industries prefer to invest in R&D for new digital technologies, rather than energy savings and emission reduction^68^. This also leads to the fact that the digital economy does not have a significant impact on the green total factor productivity of technology-intensive firms.

Fourth, technology-intensive firms tend to be more prone to the energy rebound effect. Although technology-intensive manufacturing employs a large number of green production technologies, green production technologies often lead to a reduction in output efficiency to some extent. In order to ensure overall production efficiency, an industry may choose to expand its production scale and extend its production time. This behavior offsets the reductions in energy consumption and pollutant emissions due to green production technology, making the final effects of energy saving and emission less obvious, which is reflected in the regression results, where the coefficient is not significant^71^.

The above analysis shows that there is obvious heterogeneity in the impacts of the development of the digital economy on the green total factor productivity of different categories of manufacturing industries, and hypothesis H3 holds.

## Discussion

The main regression model in this study reveals that there is a significant positive correlation between the digital economy level of manufacturing industry and green total factor productivity. This conclusion is consistent with most of the existing research results^18,21,48^, which proves that the indicator system and research method established in this paper are reasonable and persuasive. It also shows that in recent years, China's manufacturing industry has obtained high-quality development under the support of digital economy. This phenomenon is in line with Solow's assertion that "the emergence of new technology promotes the growth of TFP", which indicates that China's current economic policies are effective.

The intermediary effect analysis of this study shows that the development of digital economy mainly affects GTFP through technological progress, which is different from some existing studies. Yang and Zeng^8^, Shi and Sun^44^ believe that the improvement of digital economy on technical efficiency is more direct, and the improvement of technical progress is more complex. The reason for this may be that the focus of the two sets of indicator systems is different. The indicator system of this study is built around the manufacturing industry itself. For the industry, it is more dynamic to promote equipment renewal, because this practice is short and effective. However, there is a lag in the progress of organizational structure, employee structure, management means, marketing, etc., which is relatively difficult to adjust and slow to achieve^39^. It is reflected in the measurement model that the impact of the development of digital economy on technological progress is more obvious. The indicator system of Yang focuses on regional information infrastructure, and the improvement cycle of infrastructure is longer than that of industrial equipment, and its effect is not significant at the initial stage^38,39^. Therefore, in their model, the impact of digital economy on technological progress is not significant. This difference does not mean that one of the research conclusions is wrong, but the application scenarios of the two conclusions are different. Therefore, when formulating regional macro policies and industrial strategies, the focus should be different.

The heterogeneity analysis of this study found that the digital economy has a positive and significant impact on the GTFP of resource intensive manufacturing industry, the impact on the GTFP of technology intensive manufacturing industry is not obvious, and the impact on labor-intensive enterprises is significantly negative. This conclusion is similar to the conclusion of Hui and Yang^19^ and Zeng et al.^41^, but different from the conclusion of Cheng and Qian^18^. This study believes that the GTFP of technology intensive manufacturing industry has a positive and significant response to the digital economy. The possible reason is that their research did not consider the impact of external factors on the high-tech industry, which led to the idealization of the research conclusions. In fact, due to external factors, such as funds, policies, international relations, etc., the achievements of some existing enterprises in the process of digital transformation are indeed less than expected. This reality shows the rationality of the conclusion of this study.

## Conclusions and policy implication

Based on data that were publicly available in statistical yearbooks, this study measured the green total factor productivity of each category of enterprises in the manufacturing industry and the level of development of the digital economy during the period of 2011–2021 and explored the correlation of the latter with the former by using a panel regression model. It was found that the digital economy can enhance the green total factor productivity of the manufacturing industry as a whole, and the conclusion passed the tests of robustness and endogeneity. This means that vigorously developing the digital economy is an important path toward realizing the transformation of China's high-quality economic development. In the heterogeneity analysis, it was found that the application of the digital economy has a positive promotive effect on improvement in green total factor productivity in high-pollution manufacturing industries, while it has the opposite effect on labor-intensive manufacturing industries, and the green total factor productivity of technology-intensive manufacturing industry is insensitive to changes in the digital economy. By what path does the digital economy affect green total factor productivity? The mediating effect analysis in this study concluded that the growth of green total factor productivity brought about by green technological progress is not obvious, but because the development of the digital economy promotes green technological progress very significantly, it also raises the green total factor productivity to a certain degree. The transmission path of the digital economy (green technological progress to green total factor productivity) can play an important role. It was concluded that there is a masking effect in the path from the digital economy to green technical efficiency and green total factor productivity, i.e., although the development of the digital economy has reduced the level of green technical efficiency to a certain extent, this is not the case in the initial stage. Although the development of the digital economy has reduced the level of green technical efficiency to some extent because the rapid development of the digital economy has brought about an impact on the traditional management tools and production organization processes, but green technical efficiency is very significant for green total factor productivity, so the overall green total factor productivity is still in an upward trend. Based on this conclusion, this study argues that it is necessary to recognize that improving green technical efficiency is the fundamental way to achieve green total factor productivity, and, in the long run, it is necessary to make reasonable adjustments to the industry's management and production organization methods in a timely manner in order to quickly match the pace of the development of the digital economy.

Based on these findings, this study puts forward the following suggestions for countermeasures.

First, we should formulate a suitable path for the development of the digital economy for different types of manufacturing industries, and we must not blindly promote the digital economy reform. For labor-intensive manufacturing industries, one can appropriately reduce the speed of promotion of digital technology. Digital production technology should first be used for a small-scale pilot to prove that it can really help the industry to improve its production efficiency and reduce its energy consumption and emissions; then, it can be used for large-scale promotion. At the same time, in the promotion of digital production technologies, we should actively guide practitioners to improve their comprehensive skills and do a good job of labor diversion to alleviate the impacts of the digital transformation, which may cause unemployment and social conflicts. For highly polluting manufacturing industries, when vigorously promoting green production technology, it is also necessary to pay attention to the cultivation of practitioners' awareness of the digital economy with to the intention of giving fuller play to the positive role of the digital economy in green production, energy conservation, and emission reduction. For technology-intensive manufacturing industries, it is also necessary to pay attention to its own green development path by appropriately tilting the focus of research and development toward green production and striving to achieve the win–win goal of efficiency and emission reduction; on the other hand, it is also necessary to pay attention to the increase in pollution emissions caused by the expansion of an industry's production scale, and it is necessary for the industry's managers to boldly reform traditional management systems and rationally optimize their production plans so as to alleviate the possible energy rebound effect.

Secondly, efforts should be made to improve the quality of human resources and provide sufficient intellectual support for the development of the digital economy. The development of the digital economy is a complex systematic project that puts forward higher requirements for related practitioners; first of all, we should speed up the reform of the education system, optimize the discipline settings, and improve the layout of specialties in the digital economy so as to create a good educational environment for complex talents with excellent literacy in the digital economy. In addition, for existing groups of practitioners in the manufacturing industry, investments should be made in training their digital skills, and the industry's overall workforce should be enhanced in a short period of time in terms of digital literacy and digital operating skills in order to better release the potential for the development of the digital economy.

Third, government departments should change their working methods and should provide timely introductions and optimizations of relevant systems and regulations to provide institutional protection for the development of the digital economy. On the one hand, the government should act as a liaison and actively connect enterprises, universities, research institutions, etc. so as to break down the traditional barriers and promote the free flow of data elements, digital technology, and digital talents in different fields. On the other hand, the government can provide financial incentives and tax incentives for the development of the digital economy to reduce the costs and possible losses incurred by enterprises in the digital transformation, as well as to solve the worries of enterprises concerning innovation and trial and error; in addition, it should also legislate in a timely manner, optimize regulations, regulate the behavior of trading data elements, and crack down on data theft, privacy leakage, and other undesirable actions that seriously disrupt the normal order of the development of the digital economy.

There are some shortcomings in this study. First, the sample of this study is based on the national level, and the research method has not been extended to the regional level, so this study does not explore whether there are regional differences in the conclusions. Second, this study does not explore the unique situation of each manufacturing industry in detail, so the conclusions and suggestions of this study are slightly lacking in pertinence. In future research, the methods of this paper can be used to extend the research object to the regional level and more detailed industry level, in order to obtain more targeted policy recommendations.

## Data Availability

The data presented in this study are available from the corresponding author upon request.
